# Safety, tolerability, pharmacokinetics, immunogenicity and pharmacodynamics of 9MW1911, an anti-ST2 monoclonal antibody: results from a first-in-human phase 1 study

**DOI:** 10.3389/fphar.2025.1647816

**Published:** 2025-09-16

**Authors:** Qian Zhao, Qian Li, Yucan Wang, Li Li, Xue Zhao, Ni Wu, Diyi Fu, Danfeng Yin, Jing Feng, Zhitian Hu, Yinhan Guo, Rui Chen

**Affiliations:** ^1^ Clinical Pharmacology Research Center, Peking Union Medical College Hospital, Chinese Academy of Medical Sciences and Peking Union Medical College, Beijing, China; ^2^ Mabwell (Shanghai) Bioscience Co., Ltd., Beijing, China

**Keywords:** 9MW1911, safety, pharmacokinetics, first-in-human trial, ST2

## Abstract

**Background:**

9MW1911 is a high-affinity human IgG4 monoclonal antibody targeting ST2, the human IL-33 receptor. It may have anti-inflammatory effects by blocking the IL-33/ST2 pathway. This first-in-human trial (NCT05803902) aimed to evaluate the safety, tolerability, pharmacokinetics, immunogenicity and pharmacodynamics of 9MW1911 in healthy participants.

**Methods:**

This phase I, randomized, double-blind, placebo-controlled study enrolled 48 healthy adults. After a screening period of up to 28 days, they received a single ascending intravenous dose (ranging from 25 to 1200 mg) of 9MW1911 (n = 6 per dose) or matched placebo (n = 2 per dose). Parameters of safety, pharmacokinetics, immunogenicity and pharmacodynamics were evaluated, with follow-up visits until day 113 post-dosing.

**Results:**

9MW1911 was safe and well-tolerated across various doses. Most AEs were of mild to moderate, resolved without treatments. No dose-related AEs were observed, and the only serious AE (fetal malformation) was deemed unrelated to the study drug. No deaths or discontinuations due to AEs occurred. 9MW1911 ranging from 25 mg to 1,200 mg demonstrated a non-linear increase in exposure, while a linear PK profile was observed in the dose range from 100 mg to 1200 mg. No anti-drug antibodies were detected in any participants. Total sST2 in serum increased and stabilized at higher dose levels, demonstrating sustained target binding.

**Conclusion:**

The study demonstrates that 9MW1911 was safe and well-tolerated in healthy participants. As 9MW1911 concentrations increased,the sustained elevation of sST2 in the higher dose levels (100mg–1200 mg) suggested that the target-mediated drug disposition (TMDD) elimination became saturated, leading to the observed linear PK profile. These data support the continued development of 9MW1911 for the therapeutic use in the relevant disease.

## Introduction

Interleukin (IL)-33, a member of IL-1 cytokine family, plays a crucial role in both innate and adaptive immune response by acting as an alarmin (Liew et al., 2016). Once released, IL-33 binds to a heterodimer formed by its specific receptor ST2 and co-receptor IL-1 receptor accessory protein (Schmitz et al., 2005; Lingel et al., 2009). ST2 is expressed on various immune cells, such as group 2 innate lymphoid cells (ILC2s), T helper 2 (Th2) cell, macrophages, eosinophils and regulatory T cells (Cayrol, 2021; Molofsky et al., 2015). The IL-33/ST2 signal pathway then activates these cells, leading to the production of proinflammatory cytokines that promote immune responses, and plays a role in the pathology of various inflammatory diseases, including atopic dermatitis, asthma, chronic obstructive pulmonary disease, etc (Liew et al., 2016). Therefore, targeting IL-33/ST2 axis may serves as a promising strategy for those disease treatment.

There are two main isoforms of ST2 resulting from variable splicing, including a transmembrane receptor (ST2L) and a truncated soluble receptor (sST2) that lacks the transmembrane and cytoplasmic domains (Bergers et al., 1994; Pascual-Figal and Januzzi, 2015). Generally, sST2 is considered to act as a decoy receptor to inhibit IL-33 activity via IL-33 sequestration, thereby preventing IL-33/ST2 signaling pathway activation. However, it has also been suggested that sST2 may enhance IL-33 activity via complex formation in the lungs (Watanabe et al., 2022). 9MW1911 is a human immunoglobulin G4 (IgG4) monoclonal antibody designed to bind the extracellular domain of ST2, thereby blocking the IL-33/ST2 pathway. *In vitro* assays have demonstrated that 9MW1911 exhibited high affinity for human ST2 and effectively inhibited the binding of IL-33 to ST2, as well as the subsequent release of downstream cytokines. *In vitro* cellular assays show that 9MW1911 effectively blocks IL-33/ST2 binding with an IC_50_ of 15.95–23.15 ng/mL. Preclinical studies in cynomolugus, 9MW1911 at doses of 10 mg/kg and 30 mg/kg can significantly reduce skin swelling caused by histamine. Additionally, in a model of irinotecan-induced enteritis, 9MW1911 at 10 mg/kg can mitigate disease symptoms.

This phase I study aimed to evaluate the safety, tolerability, pharmacokinetics (PKs), immunogenicity, and pharmacodynamics (PDs) of 9MW1911 following a single ascending intravenous infusion over a broad range of doses in healthy participants.

## Method and meterials

### Study design and treatment

This was a phase I, randomized, double-blind, placebo-controlled, single ascending dose study (ClinicalTrial.gov identifier: NCT05803902) designed to evaluate the safety, tolerability, PKs, PDs, and immunogenicity of 9MW1911 in healthy Chinese adults. The study was conducted at Peking Union Medical College Hospital between December 2021 and March 2023. It was approved by the Ethics Committee of Peking Union Medical College Hospital, and was carried out in accordance with Good Clinical Practice Guidelines, the Declaration of Helsinki, and the relevant country-specific requirements. Written informed consent was obtained from all participants.

**FIGURE 1:**
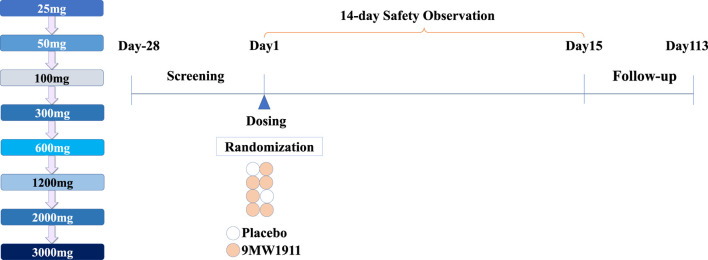
Study design.

Eligible participants were randomized in a 3:1 ratio to receive a single intravenous dose of 9MW1911 or matched placebo. Eight escalating doses were selected based on preclinical studies: 25 mg, 50 mg, 100 mg, 300 mg, 600 mg, 1,200 mg, 2000 mg, and 3000 mg. 9MW1911or placebo is diluted with 0.9% sodium chloride solution to 100 mL and intravenously infused 30 min. Each dose cohort included eight participants, and the randomization of participants to 9MW1911 or placebo was double-blind. Cohorts were enrolled sequentially, with enrollment in the next higher dose cohort initiated only after the last participant in the previous lower dose cohort had been monitored for safety and tolerability for 14 days. Dose escalation decisions were made according to predefined stopping rules. However, the 2000 mg and 3000 mg cohorts were not actually enrolled, despite the 1200 mg cohort not meeting the predefined stopping rules. This decision was primarily based on pharmacokinetic (PK) results, which suggested that the exposure at 1200 mg could provide sufficient therapeutic coverage for subsequent clinical studies, referenced data from other ST2-targeting antibodies such as CNTO7160 and Astegolimab. The AUC_inf_ of CNTO7160 is 4,415.40 ± 1,293.30 μg·day/mL following 10 mg/kg (about 600 mg for a 60 kg person) intravenous administration (Nnane et al., 2020), and the AUC_inf_ of Astegolimab is 1,000 ± 65.3 μg·day/mL and 3,750 ± 504 μg·day/mL after 210 mg and 700 mg intravenous dosing (Zhang et al., 2024). The ${\text{AUC}}_{0-\infty }$ of 9MW1911 is 5,000 ± 962 μg·day/mL at 600 mg intravenous administration and 10,100 ± 1490 μg·day/mL at 1,200 mg intravenous administration. The study enrolled healthy male and female participants, aged 18–65 years, with a body mass index (BMI) between 19.0 and 26.0 kg/m^2^. Individuals with significant allergic reactions, clinically abnormal laboratory results, or medical conditions that could affect study participation were excluded. All participants underwent an initial screening evaluation from day-28 to day-2 before randomization on day-1. On day 1, participants received their dose of 9MW1911 or placebo and were followed up through day 113 (Figure 1).

### Study outcomes and assessments

The primary outcome of this study was to evaluate the safety and tolerability of 9MW1911 following a single intravenous injection in healthy Chinese participants. Safety assessments included the treatment-emergent adverse events (TEAEs), physical examinations, vital signs, 12-lead electrocardiograms, and clinical laboratory tests, which comprised hematology, clinical chemistry, and urinalysis. The severity of TEAEs were assessed by investigators according to the Common Terminology Criteria for Adverse Events (CTCAE) version 5.0.

Secondary outcomes were the PK profile of 9MW1911 and its immunogenicity. Blood samples for PK assessment were collected predose and postdose at 0.5 h, 0.75 h, 1 h, 4 h, 8 h, 24 h, 48 h, 72 h, 96 h, 8 days, 15 days, 22 days, 29 days, 43 days, 57 days, 85 days and 113 days. An enzyme-linked immunosorbent assay (ELISA) was developed and validated to quantify 9MW1911 in human serum. Recombinant human ST2 (C-6His) was immobilised on 96-well plates as the capture antigen, followed by blocking to generate a solid-phase surface. Serially diluted calibration standards, blank matrix, quality-control samples, and unknown serum were incubated in the wells. After washing, an HRP-conjugated mouse monoclonal antibody directed against the Fc region of human IgG4 (Mouse Anti-Human IgG4 Fc-HRP) was added. In the presence of 9MW1911, a “solid-phase ST2–9MW1911–HRP-labelled antibody” complex was formed. Addition of the chromogenic substrate TMB resulted in a color change whose absorbance was directly proportional to the concentration of 9MW1911 in the sample. The lower limit of quantification (LLOQ) was 31.25 ng/mL. PK parameters included, but were not limited to, the maximum serum concentration (C_max_), area under the concentration-time curve from time 0 to the time of the last quantifiable concentrations (AUC_0-t_), area under the concentration-time curve from time 0 to infinity $\left({\text{AUC}}_{0-\infty }\right)$ , the time to reach C_max_ (T_max_), terminal half-life (t_1/2z_), volume of distribution at terminal phase (V_z_), clearance (CL) and eliminated rate constant (λ_z_).

Immunogenicity was evaluated by measuring anti-drug antibodies (ADAs), and neutralizing antibodies (NAbs) were assessed in ADA-positive serum samples. Blood samples for immunogenicity assessment were collected at predose and on days 15, 22, 29, 43, 57, 85, and 113 postdose.

As an exploratory outcome, total sST2 levels in serum were measured to assess PDs, with the blood collection time points matching those of the PK assessment. The concentration of total sST2 in serum was determined by ELISA (LLOQ = 62.5 pg/mL).

### Statistical analysis

Being a phase I trial, no formal statistical hypothesis tests were made, and analyses were done descriptively. The planned sample size of 6 active treatment participants per cohort was chosen to provide a preliminary safety and PK assessment of 9MW1911. All patients assigned to placebo were pooled together as a single group. For categorical variables, data were summarized by the number and percentage of participants in each category. Continuous variables were summarized using descriptive statistics (mean, standard deviation, median, minimum, and maximum).

Safety analyses were conducted on the safety populations, which included all participants who received at least one dose of study medication. Adverse events were coded using Medical Dictionary for Regulatory Activities (MedDRA) version 25.1. The PK population included participants who received 9MW1911 and had at least 1 measurable serum concentration post-administration. The dose proportionality for AUC_0-t_, ${\text{AUC}}_{0-\infty }$ and C_max_ was assessed by using a power model with determination of the linear regression slope with a 95% confidence interval (CI): ln (Parameter) = a+b *ln (dose)+Error. Noncompartmental analysis was to calculate the PK parameters and the analysis was done using Phoenix WinNonlin version 8.3.4. All the other statistical analyses were performed using SAS version 9.4.

## Results

### Participant demographics and baseline characteristics

331 participants were screened for eligibility, of whom 48 were randomly assigned.

All participants received one dose of 9MW1911 (n = 36) or placebo (n = 12) and completed the study. For the 36 participants who received 9MW1911, 6 participants each received 9MW1911 25, 50, 100, 300, 600 and 1200 mg.The 2000 mg and 3000 mg cohorts were not enrolled, as mentioned previously. 7 major protocol deviations were reported for 7 participants, as non-blinded personnel participated in the sample collection at the day 85 visit of the 7 participants. All protocol deviations were considered to have no effects on the study outcomes.

Baseline characteristics are shown in Table 1, and were similar across dose cohorts. The mean age was 35.1 years and 33.2 years in the placebo and the 9MW1911 group, respectively, and 66.7% of participants were male in both groups. The mean body mass index (BMI) was 23.0 kg/m2 and 22.5 kg/m2 in the placebo and the 9MW1911 group, respectively.

**TABLE 1:** Baseline demographics and characteristics of healthy participants.

Characteristics	Placebo n = 12	9MW1911						
		25 mg (n = 6)	50 mg (n = 6)	100 mg (n = 6)	300 mg (n = 6)	600 mg (n = 6)	1200 mg (n = 6)	Total (n = 36)
Age,median (min,max),years	35.0 (24,47)	29.0 (23,46)	30.5 (24,33)	37.0 (23,44)	31.0 (23,42)	36.5 (23,48)	37.5 (22,49)	31.5 (22,49)
Sex,n (%)								
Male	8 (66.7)	5 (83.3)	4 (66.7)	4 (66.7)	4 (66.7)	3 (50.0)	4 (66.7)	24 (66.7)
Female	4 (33.3)	1 (16.7)	2 (33.3)	2 (33.3)	2 (33.3)	3 (50.0)	2 (33.3)	12 (33.3)
Race,n (%)								
Chinese Han	11 (91.7)	6 (100.0)	6 (100.0)	6 (100.0)	5 (83.3)	6 (100.0)	6 (100.0)	35 (97.2)
Height, mean (SD), cm	169.0 (8.73)	167.3 (8.21)	168.0 (9.61)	167.2 (8.91)	170.0 (7.40)	165.7 (6.65)	169.7 (7.20)	168.0 (7.62)
Weight, mean (SD),kg	65.88 (9.64)	62.78 (4.66)	66.33 (8.36)	58.55 (7.11)	63.40 (7.84)	63.13 (9.17)	67.47 (10.69)	63.61 (8.11)
BMI, mean (SD),kg/m^2^	23.0 (1.7)	22.4 (0.6)	23.4 (1.1)	20.9 (1.2)	21.9 (1.5)	22.9 (1.9)	23.3 (2.2)	22.5 (1.7)

BMI, body mass index.

### Safety

All 48 participants were included in the safety analysis population. As shown in Table 2 at least one TEAE occurred in 28 of 36 participants (77.8%) who received 9MW1911 and in 8 of 12 participants (66.7%) in the placebo group. TEAEs that occurred in 10% or more of participants in the 9MW1911 group, compared with the placebo group, included aspartate aminotransferase (AST) increased (19.4% vs 0%), alanine aminotransferase (ALT) increased (16.7% vs 8.3%), blood glucose increased (16.7% vs 0%), blood triglycerides increased (13.9% vs 41.7%), and white blood cell count increased (13.9% vs 0%). There were no clear dose-related trends in the occurrence of TEAEs across the dose cohorts.

**TABLE 2:** Summary of TEAEs and TEAEs reported in ≥2 participants.

AE category	Placebo (n = 12)	9MW1911						
		25 mg (n = 6)	50 mg (n = 6)	100 mg (n = 6)	300 mg (n = 6)	600 mg (n = 6)	1200 mg (n = 6)	Total (n = 36)
Summary of TEAEs								
Participants with ≥ 1 TEAE	8 (66.7)	5 (83.3)	5 (83.3)	4 (66.7)	5 (83.3)	3 (50.0)	6 (100)	28 (77.8)
Participants with ≥ 1 TEAE of grade 3 severity^a^	0 (0)	0 (0)	2 (33.3)	0 (0)	0 (0)	0 (0)	0 (0)	2 (5.6)
Participants with ≥ 1 TESAE	0 (0)	1 (16.7)	0 (0)	0 (0)	0 (0)	0 (0)	0 (0)	1 (2.8)
Participants with ≥1 TEAE leading to discontinuation of investigational product or withdrawn from study	0 (0)	0 (0)	0 (0)	0 (0)	0 (0)	0 (0)	0 (0)	0 (0)
TEAEs reported in ≥2 participants								
Investigations	8 (66.7)	3 (50.0)	4 (66.7)	3 (50.0)	4 (66.7)	3 (50.0)	3 (50.0)	20 (55.6)
Blood triglycerides increased	5 (41.7)	0 (0)	2 (33.3)	1 (16.7)	0 (0)	1 (16.7)	1 (16.7)	5 (13.9)
Alanine aminotransferase increased	1 (8.3)	1 (16.7)	2 (33.3)	0 (0)	0 (0)	1 (16.7)	2 (33.3)	6 (16.7)
Aspartate aminotransferase increased	0 (0)	1 (16.7)	1 (16.7)	1 (16.7)	0 (0)	2 (33.3)	2 (33.3)	7 (19.4)
Blood glucose increased	0 (0)	1 (16.7)	2 (33.3)	0 (0)	1 (16.7)	1 (16.7)	1 (16.7)	6 (16.7)
Neutrophil count decreased	3 (25.0)	0 (0)	0 (0)	1 (16.7)	2 (33.3)	0 (0)	0 (0)	3 (8.3)
White blood cell count increased	0 (0)	1 (16.7)	1 (16.7)	0 (0)	1 (16.7)	0 (0)	2 (33.3)	5 (13.9)
White blood cell count decreased	2 (16.7)	0 (0)	0 (0)	1 (16.7)	1 (16.7)	0 (0)	0 (0)	2 (5.6)
Blood bilirubin increased	1 (8.3)	1 (16.7)	0 (0)	2 (33.3)	0 (0)	0 (0)	0 (0)	3 (8.3)
Gamma-glutamyltransferase increased	0 (0)	0 (0)	1 (16.7)	0 (0)	0 (0)	1 (16.7)	1 (16.7)	3 (8.3)
Blood alkaline phosphatase increased	0 (0)	1 (16.7)	1 (16.7)	0 (0)	0 (0)	0 (0)	0 (0)	2 (5.6)
Infections and infestations	0 (0)	0 (0)	0 (0)	0 (0)	0 (0)	0 (0)	3 (50.0)	3 (8.3)
Upper respiratory tract infection	0 (0)	0 (0)	0 (0)	0 (0)	0 (0)	0 (0)	3 (50.0)	3 (8.3)
Skin and subcutaneous tissue disorders	0 (0)	1 (16.7)	0 (0)	0 (0)	0 (0)	0 (0)	1 (16.7)	2 (5.6)
Rash	0 (0)	1 (16.7)	0 (0)	0 (0)	0 (0)	0 (0)	1 (16.7)	2 (5.6)

The table details TEAEs, by system organ class and preferred term, and TEAEs, were coded with the Medical Dictionary for Regulatory Activities (MedDRA), version 25.1. Data are number (%) of participants with a TEAE, participants are counted once for each system organ class and preferred term regardless of the number of events.

^a^ The TEAE ‘foetal malformation’, reported in the participant’s female partner, was not included in the severity grade analysis.

TEAE, treatment-emergent adverse event; TESAE, treatment-emergent serious adverse event.

The majority of TEAEs were grade 1 or 2 in severity and resolved completely without treatment. One participant at the 1200 mg dose developed a grade 2 rash on the eighth day after 9MW1911 administration, requiring 99 days of oral corticosteroids, and several days of oral antihistamines. This event finally resolved and was considered by the investigator to be related to the study agent.

Only four grade 3 TEAEs were reported, occurring in two participants within the 50 mg cohort. One participant experienced a grade 3 blood triglycerides increased, which remained unresolved before the participant was lost to follow-up. Another participant developed grade 3 AST increased, ALT increased, and gamma-glutamyltransferase increased, all of which were not associated with any clinical symptoms, and resolved without treatment.

The only serious TEAE was foetal malformation, reported in the female partner of a participant in the 25 mg cohort. This event was considered to be due to the advanced maternal age and not to a drug-related cause. No TEAEs led to discontinuation or participant withdrawal from the study and no deaths were reported. There were no clinically significant findings in vital signs (systolic blood pressure, diastolic blood pressure, pulse rate, respiratory rate and body temperature) results or electrocardiogram measurements.

### Pharmacokinetics

**FIGURE 2:**
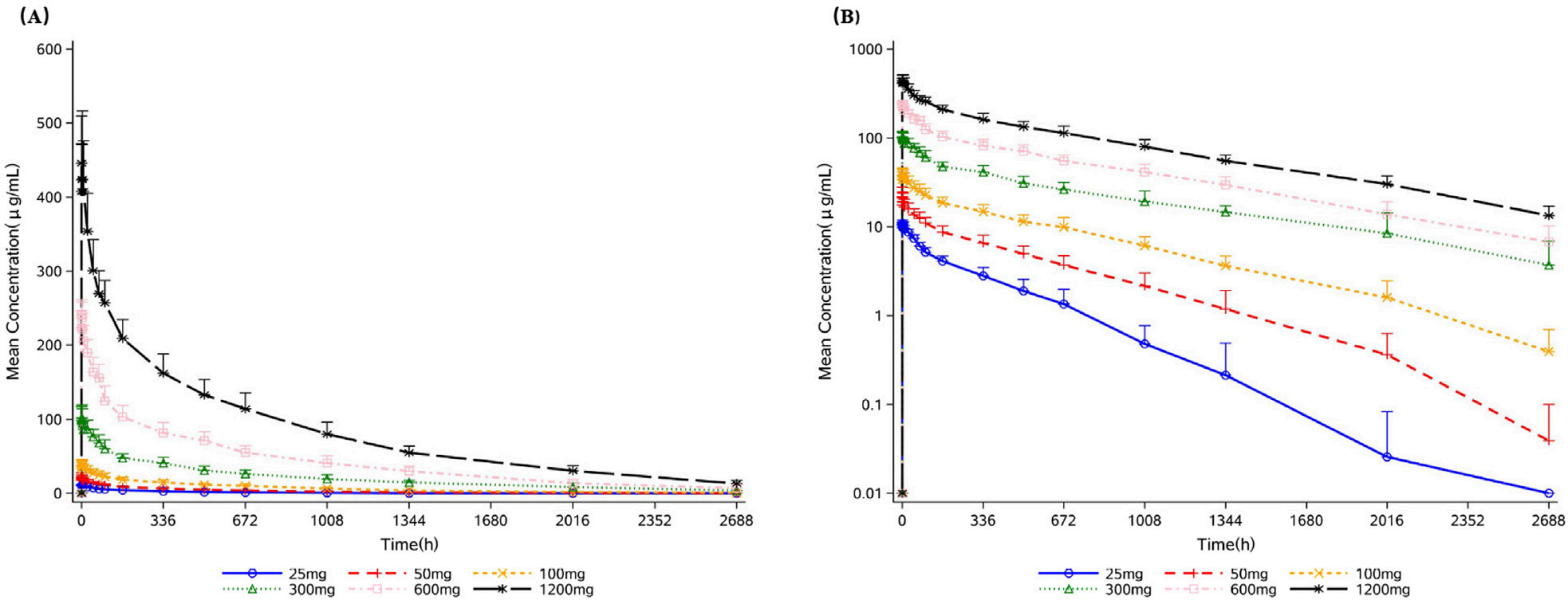
Mean (standard deviation) serum 9MW1911 concentration-time curves following a single intravenous dose of 25 mg, 50 mg, 100 mg, 300 mg, 600 mg, and 1200 mg 9MW1911. **(A)** Linear scale; **(B)** Semi-log scale.

All participants who received 9MW1911 were included in the PK analysis. Mean concentration-time curves of 9MW1911 following a single intravenous dose of 25 mg, 50 mg, 100 mg, 300 mg, 600 mg, and 1200 mg 9MW1911 are shown in Figure 2, and the PK parameters are summarized inTable 3. The maximum concentrations of 9MW1911 in serum were achieved immediately following intravenous administration, and the median T_max_ range was 0.75–1 h for the 25–1200 mg 9MW1911 doses. The mean t_1/2z_ ranged from 9.12 to 31.7 days, and has a trend to increase with increasing 9MW1911 doses, but was similar across higher-dose groups: 26.1 days (300 mg), 25.6 days (600 mg) and 31.7 days (1200 mg). The mean V_z_, CL and λ_z_ values ranged from 2,760–5,560 mL, 5.06–9.29 mL/h and 0.92–3.45 × 10^−3^/h, respectively.

**TABLE 3:** Mean (SD) pharmacokinetic parameters of 9MW1911 following a single intravenous dose in Chinese healthy participants.

Parameters	25 mg (n = 6)	50 mg (n = 6)	100 mg (n = 6)	300 mg (n = 6)	600 mg (n = 6)	1,200 mg (n = 6)
C_max_ (μg/mL)	11.0 (0.94)	21.7 (6.25)	38.0 (8.19)	105 (17.6)	243 (19.3)	458 (79.6)
AUC_0-t_ (day·μg/mL)	116 (29.3)	297 (71.5)	749 (176)	2,280 (539)	4,720 (821)	9,440 (1,380)
AUC_0–∞_ (day·μg/mL)	117 (30.1)	303 (70.9)	760 (185)	2,460 (739)	5,000 (962)	10,100 (1,490)
AUC_extrap_ (%)^a^	1.33 (0.85–1.89)	1.35 (0.68–6.01)	1.05 (0.32–2.75)	4.02 (1.50–15.4)	5.47 (1.31–8.64)	6.72 (3.51–8.07)
T_max_ (h)^a^	1.00 (0.75–4.00)	0.75 (0.75–4.00)	1.00 (0.75–1.00)	0.88 (0.50–4.00)	0.75 (0.75–1.00)	0.75 (0.75–4.00)
t_1/2z_ (day)	9.12 (3.08)	15.1 (3.11)	18.4 (3.52)	26.1 (11.0)	25.6 (6.20)	31.7 (3.87)
V_z_ (mL)	2,760 (362)	3,660 (841)	3,580 (673)	4,460 (727)	4,430 (591)	5,560 (1,030)
CL (mL/h)	9.29 (2.15)	7.19 (1.72)	5.76 (1.37)	5.46 (1.56)	5.21 (1.30)	5.06 (0.77)
λ_z_ (×10^–3^/h)	3.45 (1.09)	2.01 (0.53)	1.61 (0.30)	1.28 (0.50)	1.19 (0.33)	0.92 (0.11)

^a^ AUC_extrap_ and T_max_ presented as median (minimum–maximum).

SD,standard deviation; C_max_, maximum serum concentration; AUC_0-t_, area under the concentration-time curve from time 0 to the time of the last quantifiable concentration; AUC_0–∞_ area under the concentration-time curve from time 0 to infinity; AUCextrap, extrapolated portion of AUC_0–∞_; T_max_, the time to reach C_max_; t_1/2z,_ terminal half-life; V_z_, volume of distribution at terminal phase; CL,clearance; λ_z_, eliminated rate constant. AUC_extrap_ = [(AUC_0–∞_-AUC_0-t_)/AUC_0–∞_]×100%.

The 9MW1911 exposure (defined by C_max_, AUC_0–t_ and AUC_0–∞_) increased in a dose-dependent manner. Dose proportionality analysis were conducted, and the slope for dose proportionality between 25 mg and 1200 mg was 0.967 (95% CI 0.920–1.013) for C_max_, 1.127 (95% CI 1.070–1.184) for AUC_0–t_, and 1.141 (95% CI 1.082–1.201) for AUC_0–∞_, indicating that exposure increased in a more than dose-proportional manner. Dose proportionality was further evaluated stepwise from the lowest dose, and a linear PK profile was observed in the dose range from 100 mg to 1200 mg, with the slope was 1.020 (95% CI 0.941–1.099) for C_max_, 1.028 (95% CI 0.937–1.120) for AUC_0–t_, and 1.047 (95% CI 0.944–1.149) for AUC_0–∞_.

### Immunogenicity

None of the participants developed ADA response.

### Pharmacodynamics

**FIGURE 3:**
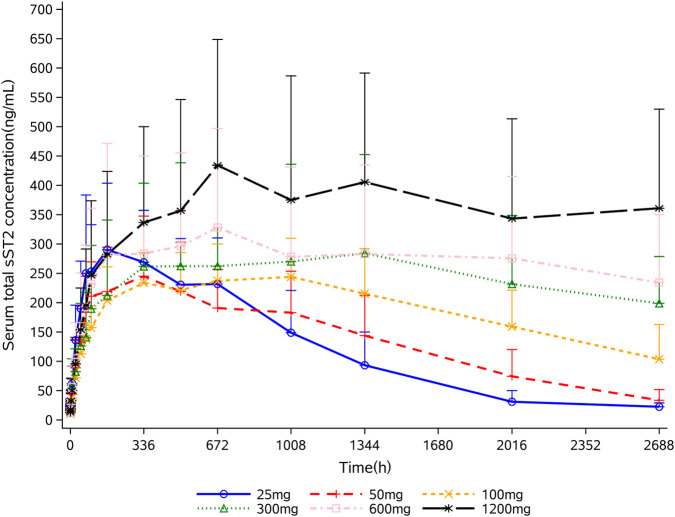
Target binding of 9MW1911 assessed by total sST2 in serum. Data are shown as mean (standard deviation). sST2 = soluble ST2.

All participants who received 9MW1911 were included in the pharmacodynamic (PD) analysis. Mean concentration-time profiles are shown in Figure 3. Serum total sST2 increased following single intravenous administration of 9MW1911, and its level increased with increasing doses. Maximum concentrations were observed at approximately day 8 to day 29, and ranged from 244 to 405 ng/mL. Total sST2 in serum increased and remained stable over an extended period at higher dose levels, demonstrating sustained target binding.

## Discussion

This was the first-in-human randomized, double-blind, placebo-controlled, single ascending dose study of 9MW1911, a monoclonal antibody targeting the IL-33/ST2 pathway, evaluating its safety, tolerability, PKs, immunogenicity and PDs in healthy Chinese participants. The study demonstrated that 9MW1911 was safe and well tolerated over a dose range from 25 mg to 1,200 mg. 9MW1911 showed a linear serum PK profile in the dose range from 100 to 1200 mg. None of the participants developed ADA response, and target binding was demonstrated with the PD result, which showed elevated total sST2 concentration.

No clear dose-related trends in TEAEs were observed across the dose cohorts, and no TEAEs led to study discontinuation, participant withdrawal, or death. Some TEAEs in the Investigations SOC were slightly more frequent in the 9MW1911 groups than in the placebo groups, such as AST increased, ALT increased, blood glucose increased and white blood cell count increased. Subsequent studies will further monitor these parameters to assess their incidence in patients. Of note, one participant in the low-dose group (50 mg) experienced three grade 3 TEAEs, which were elevations in three parameters related to liver function. Such elevations were not observed in the preclinical studies, and the mechanism remains unclear. Firm conclusion of the relationship between liver enzyme elevation and the study agent could not be drawn in this study. It will be necessary to identify the influencing factors of liver enzyme elevations and determine whether they are drug-related in further studies. A grade 2 rash occurred in the 1200 mg cohort should also be noted, as it required long-term treatment and was considered related to the study agent. Due to the small sample size, the incidence of this event could not be determined, but it will be further evaluated in the follow-up studies.

Following intravenous administration, 9MW1911 showed a mean t_1/2z_ of 9.12–31.7 days across 25–1200 mg doses, with similar values in higher-dose groups. The half-life of 9MW1911 seems longer than that reported for the other ST2-targeting antibodies like CNTO7160 (t_1/2_:2.67–20.55days) following a single intravenous dose (0.01–10 mg/kg)in healthy adults (Nnane et al., 2020). This study demonstrated the long half-life of 9MW1911, supporting long dosing intervals in subsequent studies.

A single intravenous dose of 9MW1911 ranging from 25 mg to 1,200 mg demonstrated a non-linear increase in exposure, while a linear PK profile was observed in the dose range from 100 mg to 1200 mg. This result may suggest that 9MW1911 exhibits target-mediated drug disposition (TMDD), where the drug binds with high affinity and specificity to cell receptors, leading to internalization and degradation, and affects the disposition of drug in the body (Levy, 1994; Mager and Jusko, 2001). At lower drug concentrations, a significant proportion of the drug binds to the target receptor, and the TMDD is considered as the major route of elimination, resulting rapid clearance at lower doses (Ryman and Meibohm, 2017). As the drug concentration increases, due to the limited availability of the target receptors, the drug becomes sufficient to saturate the TMDD elimination pathway, leading to linear PK profile at higher doses. It should be noted that the reported half-life and AUC_inf_ may be inaccurate in the lower-dose level owing to TMDD.

Antibodies targeting the IL-33/ST2 pathway aim to treat relevant diseases by reducing immune-mediated inflammation. Phase 2 studies have shown their potential in treating chronic obstructive pulmonary disease (COPD) (Rabe et al., 2021; Yousuf et al., 2022), and several phase 3 studies are currently underway to further assess the efficacy and safety (NCT05595642, NCT04701983, NCT05166889). The current study was the first clinical study to evaluate the safety, tolerability, PKs, immunogenicity and PDs of 9MW1911, and the acceptable safety profile supports further evaluation of the efficacy and safety of 9MW1911 in a phase 1b/2a COPD study (NCT06175351).

There are some limitations in this study. Firstly, total sST2 levels in serum were measured to assess PDs, including both free sST2 and sST2 bound to 9MW1911. The results showed increased total sST2 levels following 9MW1911 administration. This effect is probably due to the binding of sST2 to 9MW1911, which may prolong its half-life, and it can indirectly demonstrate the target binding. However, the levels of free sST2 can reflect the status of 9MW1911 binding to sST2 and determine whether the binding is saturated, making it a more sensitive PD marker. We did not measure free sST2 levels, which is a limitation of this study. Secondly, we did not measure downstream biomarkers of IL-33/ST2 signaling pathway. Given the lower baseline levels of these biomarkers in healthy individuals compared with patients (Akdis et al., 2020), changes following treatment were expected to be modest, limiting the interpretability of PD effects. These will be further explored in patient studies.

## Conclusion

The study demonstrates that 9MW1911 was safe and well-tolerated in healthy participants, with a linear serum PK profile across higher dose levels. Sustained elevation of total sST2 levels suggested target binding. These data support the continued development of 9MW1911 for the therapeutic use in the relevant disease.

## Data Availability

The original contributions presented in the study are included in the article, further inquiries can be directed to the corresponding author.
